# The Challenges of Multiculturalism in Belgian Emergency Services: An Exploration of Emergency Physicians’ Experiences, Attitudes and Intercultural Competence

**DOI:** 10.7759/cureus.77930

**Published:** 2025-01-24

**Authors:** Adèle Vincent, Erdem Yılmaz, Frédéric Thys

**Affiliations:** 1 Emergency Medicine, Faculty of Medicine, Cliniques Universitaires Saint-Luc, Catholic University of Louvain, Brussels, BEL; 2 Social and Cultural Psychology, Faculty of Psychology and Educational Sciences, KU Leuven, Leuven, BEL; 3 Acute Medicine, Grand Hospital of Charleroi (GHdC), Charleroi, BEL

**Keywords:** cross-cultural communication, cultural adaptation, cultural differences, cultural sensitivity, emergency medicine, immigration, intercultural competence, migrant health, multiculturalism

## Abstract

The increasing cultural diversity in Belgium necessitates an understanding of the challenges faced by emergency physicians in treating migrant patients. Cultural differences influence how patients experience and communicate their symptoms while migration often alters individuals' ways of being and relating to others. Despite the critical importance of these issues, no prior studies to the best of our knowledge have examined the culture-specific challenges faced by emergency physicians in Belgium. This exploratory study aims to address this gap by investigating these challenges and evaluating physicians' levels of cultural sensitivity and self-reported intercultural competence.

An online survey was conducted between September and December 2023 with 50 emergency physicians (62% female, 36% male, M_age_​ = 39.88) from Brussels and Walloon regions in Belgium. Our analyses demonstrated that physicians expressed strong interest in treating migrant patients, yet 60% reported experiencing communication barriers. A majority pointed to the absence of 24/7 translation services (98%), minority language documentation (82%), and universal pictograms (90%) to facilitate communication. Furthermore, intercultural competence was positively associated with age and higher among physicians in urban areas. However, older physicians were more likely to view cultural adaptation as the responsibility of patients, whereas younger physicians emphasized the importance of understanding patients' sociocultural backgrounds and migration histories. Notably, self-reported intercultural competence did not correlate with attitudes on cultural adaptation or the importance assigned to patients’ backgrounds.

These findings highlight systemic barriers to equitable care and reveal inconsistencies in physicians’ beliefs about cultural adaptation. The absence of correlations between intercultural competence and cultural sensitivity measures underscores the limitations of self-reported assessments. Addressing these challenges requires structural interventions, such as multilingual documentation, universal pictograms, and translation services, to support physicians and improve patient outcomes. As such, the study provides initial insights into the Belgian context and calls for further exploration of organizational and cultural changes to foster inclusive emergency care.

## Introduction

Ensuring constant accessibility for everyone, emergency services have become sites of evident multiculturalism in the increasingly cosmopolitan societies of the 21st century [1]. Belgium, as a developed European country, faces similar trends with a growing immigrant population [2]. Adding to this complexity is Belgium's unique cultural and institutional context, characterized by three linguistic communities; French-speaking, Dutch-speaking, and German-speaking, resulting in a high degree of institutional complexity. Migration patterns and motives also vary across regions. Approximately 10% of Belgium’s population holds foreign nationality, a figure that rises to 35.3% in the Brussels-capital region [2]. This demographic diversity naturally extends to the healthcare system, raising important questions about how it accommodates the language, cultural, and habit-based needs of a diverse patient population. Emergency departments, often the frontline of medical care, are at the forefront of these challenges, particularly given their high patient turnover and frequent overcrowding. As a result, the interactions between migrant patients and the physicians within emergency departments, are becoming more and more imbued with challenges posed by multiculturalism. Hence, understanding how these dynamics play out in such high-pressure settings is of utmost importance.

The complexity of these questions stems from the multifaceted nature of culture itself, which encompasses interwoven notions and practices that adapt to rapidly changing societal conditions, such as global conflicts, pandemics, and potential climate-change-induced migration [3]. Given this increasing diversity, addressing these challenges is crucial to ensuring equitable access to quality healthcare for all. For example, in 2011, the Belgian Ministry of Health initiated a study to provide recommendations for the care of migrant populations [4]. Although some of these recommendations were adapted to and incorporated within the Belgian healthcare system, their widespread practical application still remains inconsistent and limited. Meanwhile, challenges persist for both physicians and patients. Several studies conducted in Belgium have highlighted a paradox in the health outcomes of Belgian minority populations: while they experience lower mortality rates, reduced prevalence of certain cancers, and lower alcohol consumption [5], they also face higher rates of psychiatric disorders [6], type 2 diabetes [7], and self-reported poorer health [8]. Additionally, these populations encounter significant barriers to accessing preventive and specialized care [9].

Given these disparities, along with certain others, it becomes important to consider the cultural backgrounds of the patients that come to the emergency departments while providing care [10]. Adapting communication styles and medical approaches to account for cross-cultural differences can enhance patient care. Steps in this direction have already been taken in the United States, where the concept of understanding patients' cultural influences on health emerged in the 1970s [11]. From the idea of comprehending patients' experiences and trying to understand how they perceive illness emerged the conviction that patients' culture greatly influences how they manage their health [11]. As such, acknowledging patients' cultural backgrounds has been integrated into medical care, and efforts have been made to train doctors in multiculturalism [12]. Many medical curricula now include specific training in intercultural communication, understanding other cultures, and traditional medicine [13-15]. Translation services are also available in some states and special consultations are organized for patients from cultural minorities [16,17]. However, disparities remain significant [4,12,18], confirming that achieving equality in healthcare is still a work in progress.

Nevertheless, this still seems to be an important endeavor, and an even necessary one. The importance of inclusive care is further underscored by Belgian legislation. Article 5 of The Patients' Rights Act of August 22, 2002, explicitly states that all patients are entitled to quality care that respects their dignity and autonomy, without distinction based on cultural or socioeconomic background [19]. What seems evident from a moral and ethical perspective is therefore already enshrined in Belgian law. Hence, Physicians and policymakers must thus ensure that emergency care is equitable and accessible to all, regardless of their cultural and/or socioeconomic backgrounds.

Culture and medicine

In broad strokes, culture is described as a framework for understanding human behavior [20]. It encompasses the emergent properties inherent within various social and structural systems, including ethnic, socioeconomic, or gender groups, which are defined by shared symbols, meanings, and normative beliefs [20-22]. These properties are transmitted across generations [23], yet remain dynamic and subject to change [24]. Cultural differences not only influence individuals’ ways of defining themselves in relation to others [25] but also the way they experience and communicate their illnesses [26]. Within the scope of this study, we limit our understanding of culture to the different sets of beliefs and practices people might entertain based on ethnic and sociocultural differences.

Indeed, cultural differences influence individuals’ perceptions of illness, shaping their "explanatory models" and responses to health issues [26]. Medical anthropologists, such as Kleinman and colleagues, differentiate between "disease," which refers to the biomedical dysfunction of an organ, and "illness," which describes the subjective experience of that dysfunction [26]. Kleinman and colleagues further highlight a recurring conflict that caregivers may have with their patients, namely the biomedical vision overriding the patient's real concerns when seeking medical care [26]. The experience of illness, defined by personal differences and socioeconomic factors, is also largely influenced by culture. From a young age, we learn to be "sick" according to our family's cultural codes and practices. These factors shape our perception of discomfort caused by illness and the appropriate response, which defines an individual's unique "explanatory models" of illness [26]. It thus seems necessary to make a compromise between the biomedical vision (i.e., the "disease," or the "cure") and the various explanatory models of illness held by our patients (the "care") [26]. Otherwise, ignoring patients' cultural backgrounds and the misunderstandings that follow can lead to significant healthcare disparities [4,12,18].

Implications for emergency medicine

In emergency departments, which are generally structured for maximum efficiency to meet their objectives such as 24/7 accessibility and safety, the care of patients from cultural minorities presents an additional challenge [11]. In unplanned healthcare settings, most caregivers tend to adopt the dominant culture's perspective [27], making cultural mismatches and consequent miscommunications more likely. The ensuing misunderstandings can lead to recurring conflicts, frustration, or even aggression [11,28] while also creating insecurity and prolonging patient suffering [11]. Additionally, communication in acute care settings is particularly critical and challenging, and hence, special attention must be warranted in these settings [29,30].

Given these conditions, it is logical, if not intuitive to think that emergency services face difficulties due to this cultural disconnect, causing significant stress on both ends of the care-for the patients as well as the caregivers. Indeed, improving intercultural communication and reception practices could not only enhance the quality of care for patients from cultural minorities but also benefit the general population at large [4]. Furthermore, this could also improve the overall well-being and the working conditions of healthcare providers [18]. As such, the importance and necessity of increased cultural sensitivity and awareness in emergency medicine seems evident. However, to our knowledge, there are only a few studies evaluating the cultural sensitivity, opinions, and orientations of healthcare professionals in treating patients from cultural minorities and/or migrant patients within the continental European context. While these studies are rare, they highlight the importance of establishing cultural training to improve healthcare accessibility and quality [31-35]. Hence, it becomes crucial to address the gaps in the literature and come up with further studies enquiring into the issue of multiculturalism in European healthcare settings.

The current study

Recognizing the significant influence of culture on patients' illness experiences and their interactions with caregivers, alongside the existing gap in European literature, this study provides an initial evaluation of the cultural sensitivity of Belgian emergency physicians. It also examines how these physicians approach and interpret issues related to the care of migrant patients in unplanned care settings. With the ultimate aim of improving healthcare accessibility and working conditions for healthcare professionals in emergency departments, we opted to conduct an initial exploratory study within the context of the French-speaking emergency departments in Belgium. In Belgium, to become an emergency specialist, one has to complete six years of additional training upon obtaining a medical degree, which itself used to be a seven-year long program but was shortened to six years from the academic year 2012-2013 onwards. For our study, we decided to include both emergency specialists and assistant emergency specialists who are still in training to become emergency specialists. As such, we aimed to have an added degree of comparison regarding the seniority of the physicians.

There were several questionnaires available within previous literature to evaluate the intercultural competence of medical students, doctors, or nurses [36,37]. Each one of these questionnaires has its own advantages and disadvantages [38] and is adapted to a specific socio-cultural context. Nonetheless, most of these surveys are conceptualized in English-speaking contexts to assess the returns associated with multicultural training. Since this type of training does not yet exist in Belgium, such previous literature proved to be of limited relevance to our work.

As a result, the questionnaire we found to be most appropriate for our research goals was developed by Hudelson and colleagues [34,35] to assess the intercultural competence and attitudes of physicians in Switzerland. We found their questionnaire particularly relevant to our study, as it does not directly assess multicultural training, which, as noted earlier, is not available in Belgium, but instead evaluates physicians' interest in and perspectives on treating patients from cultural minority groups. Additionally, this questionnaire enables us to see the associations between respondents' demographic characteristics and their reported levels of cultural sensitivity. In order to adapt this questionnaire to the Belgian as well as the emergency medicine context, we removed one category (i.e., clinical vignettes) and replaced it with another subscale that incorporated items pertaining specifically to the emergency service working environments.

To be precise, with this study our aims are to explore physicians’ beliefs, attitudes, and/or opinions on the care of migrant patients and the key challenges posed thereby, the amenities and facilities at disposal while attending migrant patients, their self-assessed levels of intercultural competence, with whom lies the responsibility for cultural adaptation, and the importance of knowing patients’ cultural backgrounds and their histories of migration. By tapping into these different domains, we more broadly aim to raise awareness concerning multiculturalism and the challenges it poses for emergency physicians. The data collected within the framework of this study is also intended to serve as an initial point of reference to monitor any future actions or changes regarding multiculturalism within the Belgian context.

The findings of the study that constitute this article were previously presented as a meeting abstract at the 2024 BeSEDiM National Congress at Ostend on May 31, 2024.

## Materials and methods

Participants and procedure

Prior to the distribution of the questionnaire, ethical approval was obtained from the ethics committee of St. Luc University Clinics. The questionnaire was prepared and distributed through LimeSurvey, and the data was stored on the servers of the Catholic University of Louvain. All throughout the process we ensured complete anonymity of all the participants. Consequently, emails with a link to the questionnaire were sent to emergency physicians (including assistants) in hospitals in Wallonia and Brussels, between September 2023 and December 2023. All respondents were asked to provide consent before participation, and they could withdraw their consent at any moment of participation. The participants were asked to respond to all questions with the care of migrant patients in mind. Within the questionnaire, the term "migrant" was defined as someone born and raised outside of Belgium regardless of status (e.g., economic migrant, asylum seeker, refugee, etc.), and hence, designated what is broadly referred to as a first-generation immigrant in social-scientific literature.

Consequent to data collection and cleaning, our final sample included all the emergency physicians and assistant emergency physicians who completed the survey (N=50). Given the specificity of our target sample (i.e., French-speaking emergency physicians in Brussels and Wallonia) we deemed this sample size representative of an exploratory study and thus carried out our analyses with this data. Of the participants from whom we received complete responses, 62% identified as female, 36% identified as male, and 2% did not specify their gender. The age of the participants ranged between 25 and 62 (Mean age=39.88, SD=11.5). Among all the participants, 64% were emergency physicians, 36% were assistant physicians still in training to become emergency specialists, 92% were Belgian nationals, and 82% worked in hospitals located in an urban area. Table 1 provides a summary of participant characteristics by group.

**Table 1:** Participant characteristics by group. Summary of demographic and professional differences between emergency physicians and emergency assistants. It displays the mean ages for emergency assistants and specialists separately: the mean age for specialists is 45.91, while for assistants, it is 29.17. The rest of the data has been represented as N and % with respect to the total sample, N=50.

	Emergency specialists (N,%)	Emergency assistants (N,%)
N	32, 64%	18, 36%
Mean_age_	45.91	29.17
Nationality		
Belgian	30, 60%	16, 32%
Other	2, 4%	2, 4%
Working area		
Urban	27, 54%	14, 28%
Rural	5, 10%	4, 8%

Materials 

Our adapted questionnaire constituted a survey that consists of 84 questions in total, tapping into the five domains/dimensions of inquiry that pertain to the objectives of our exploratory study. All materials were originally developed and administered in French, and therefore, no translations were made. The complete questionnaire can be seen in Appendix A and Appendix B within the online supplementary materials (OSM).

Experiences and Challenges of Multiculturalism in the ER

The first domain of inquiry was physicians’ general experiences with migrant patients and the frequency of commonly encountered challenges posed by cultural differences in the care of migrant patients in daily practice. Here, we had four general questions on their experiences with migrant patients, gauging the estimated prevalence of migrant patients among all the patients they attend, the frequency and severity of the difficulties they face while caring for migrant patients, and the level of interest they have specifically towards caring for migrant patients.

We also included seven questions specific to their experiences of caring for migrant patients within the emergency room context. These included items such as; "Do you think that being a migrant can lead to conflicts in the emergency room?”; “Do you think that emergency departments see more migrant patients than other medical specialties?”; “Do you think it would be necessary to structure emergency services in such a way that they can better accommodate migrant patients?”, scored on a 5-point Likert scale ranging from 1=totally disagree to 5=totally agree. We also provided the participants with a list of 15 commonly encountered challenges and asked them, for each factor, to indicate whether it represents a frequent problem they face. The list included items such as “migrant patient's health beliefs that conflict with medical knowledge”, “physician bias or prejudice towards migrant patients”, “unclear complaints expressed by the migrant patient”, etc. Again, each item was scored on a 5-point Likert scale ranging from 1=totally disagree to 5=totally agree based on how frequently the physicians perceived encountering them.

Amenities Available

The second domain of inquiry was the amenities and facilities at the disposal of the physicians in addressing the challenges they face in the care of migrant patients. Here, we asked participants whether, in the emergency services where they worked, they had access to professional translation services, and if yes, whether these services were available on a 24/7 basis, documentation in languages other than the three official languages of Belgium (i.e., French, Dutch, or German), and universal pictograms to facilitate communication with patients who do not speak any common languages with the caretakers.

*Self-Assessed Intercultural Competence*.

Thirdly, we measured physicians’ self-assessed levels of intercultural competence in 14 clinical tasks. Here we utilized a 14-item subscale (α=0.70) that asked the participants whether they considered themselves to be competent in carrying out certain tasks regarding the care of migrant patients, scored on a 5-point Likert scale ranging from 1=totally disagree to 5=totally agree. These items referred to other tasks such as taking a psychosocial history of the patient, breaking bad news to a migrant patient, referring an undocumented migrant patient to the appropriate medical and social services, and discussing the patient's religious preferences and constraints regarding their treatment.

Responsibility for Cultural Adaptation

The fourth domain we explored was the beliefs and opinions of physicians regarding with whom the responsibility to adapt lies as far as the cultural norms involved are concerned (i.e., the institutional cultures becoming more inclusive to accommodate the migrant patients’ cultural norms vs. the migrant patient adapting to the prevalent culture of the setting in which the healthcare institution is situated). This was measured through a scale (5 items, α=0.75) scored on a 7-point Likert scale, where 1 referred to the institution/physician accommodating the migrant patients’ cultural norms and 7 referred to the migrant patients adapting to the majority culture. Here an example item read: “When the patient expresses a preference to be treated by a male or female doctor,” with 1 indicating that the hospital has to allow the patient to choose the sex of the physician, and 7 indicating that the patient has to accept to be treated by the physician assigned by the hospital regardless of the sex of the physician assigned.

Knowledge of Patients’ Background

Our fifth principal domain of investigation was whether emergency physicians deemed it important to have a certain degree of acquaintance with certain aspects of the sociocultural background as well as the migration history of the migrant patient. This was measured through a scale (9 items, α=0.86) scored on a 5-point Likert scale ranging from 1=totally disagree to 5=totally agree, and included items such as knowledge of the patient's ability to read and write, knowledge of the patient's religious or spiritual beliefs, and the general knowledge of the history and culture of the migrant patient's country of origin.

Data analysis strategy

Given the lack of previous studies within the Belgian context, we opted for an exploratory analytical approach. This seemed relevant, especially due to the absence of comparative data on the care of migrant patients in Belgian emergency services. To this end, we performed exploratory descriptive analyses, linear regression analyses, and ANOVAs to examine relationships and differences between variables. All analyses were conducted using IBM SPSS Statistics 30, and the following statistical parameters are reported: β, the standardized regression coefficient, indicates the strength and direction of the relationship between predictors and the dependent variable; F, the ANOVA test statistic, evaluates whether group means significantly differ, with larger values reflecting greater variability between groups relative to within-group variability; t, the test statistic for regression coefficients, determines whether a predictor significantly contributes to the model, with larger absolute values indicating stronger contributions; and p-values, which reflect the probability of observing the data under the null hypothesis. Results are considered statistically significant at p<0.05 unless otherwise specified.

## Results

Regarding the general experiences of multiculturalism in the ER and the challenges thereof, participants estimated that migrant patients represented around 15.02% (SD=14.83) of the patients they encountered in their practice. Our analyses showed that the majority of respondents expressed a strong interest (M=4/5, SD=1.15) in caring for migrant patients, yet only 10% of them reported not having significant problems during the care of migrant patients. Indeed, 66% of the participants reported that the difficulties they encountered while communicating with these patients were severe, and 6% reported that these difficulties were very severe. On average, participants perceived that migrant patients were more likely to seek care from emergency specialists compared to other medical specialties (M=4.14, SD=0.134). They also reported that migrant patients tend to return to emergency services more frequently (M=3.48, SD=0.172). On average, our respondents reported having experienced a consultation with a migrant patient where they did not understand the reason for the visit (M=3.88, SD=0.150), leading to uncertainty about the complaints throughout the consultation.

Emergency physicians most frequently identified the following three difficulties as the number one challenge regarding the care of migrant patients in the emergency services: the patient’s lack of proficiency in French, the vague or imprecise complaints of the patients, and patients' unrealistic expectations from the physicians.

**Figure 1:**
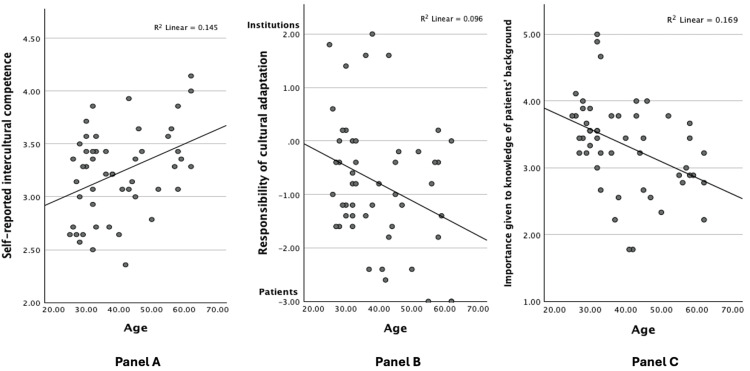
Scatter plots for physician’s age vs. self-reported intercultural competence. Importance given to the knowledge of migrant patients’ sociocultural backgrounds, and attitudes concerning the responsibility of cultural adaptation. Lines represent best-fit lines for linear regression analyses. Panel A: Self-reported intercultural competence increases with age, β=0.381, t=2.852, p=0.006. Panel B: Older physicians tend to think more that it is the migrant patients’ responsibility to adapt to the cultures of the institutions, β=-0.310, t=-2.256, p=0.029. Panel C: Younger physicians place greater importance on knowledge of patients' backgrounds, β=-0.411, t=-3.125, p=0.003.

Regarding the resources available in emergency services to facilitate effective communication with migrant patients, 98% of the participants reported a lack of 24/7 translation services, and 82% stated that they did not have documentation available in languages other than the official languages of Belgium (i.e., Dutch, French, and German). Additionally, 90% of the participating physicians reported the absence of basic communication aids such as universal pictograms.As far as the self-reported intercultural competence levels were concerned, our linear regression analyses showed that self-reported intercultural competence increases with age, β=0.381, t=2.852, p=0.006 (see Figure 1A). We found no significant association with the perceived frequency of encounters with migrant patients, β=0.175, t=1.234, p=0.223. 

Additionally, our ANOVAs showed greater self-reported intercultural competence levels among emergency physicians working in urban areas (vs. those working in rural settings), F(1, 49)=4.222, p=0.045. We found no significant differences in self-reported intercultural competence levels for gender F (2,49)=0.466, p=0.631, having an international professional experience, F(1, 49)=1.663, p= 0.203, or for professional status (i.e., specialist physician vs. assistant specialist), F(1, 49)=1.478, p=0.230.

Regarding physicians’ beliefs as to with whom lies the responsibility for cultural adaptation, our linear regression analyses showed that with increasing age, physicians tend to believe more that it is the migrant patients’ responsibility to adapt to the dominant culture of the settings in which the healthcare institutions are situated, β=-0.310, t=-2.256, p=0.029 (see Figure 1B). We found no significant associations with the perceived frequency of encounters with migrant patients, β=-0.009, t=-0.063, p=0.950.

**Figure 2:**
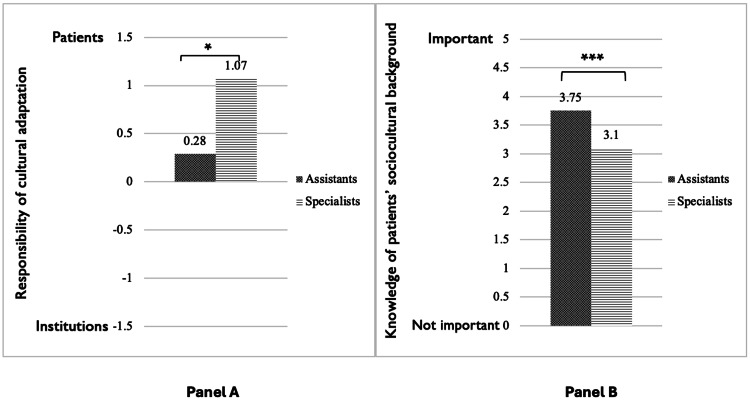
Specialists’ and assistants’ opinions on cultural adaptation and the knowledge of patients’ backgrounds. Bars represent group mean differences analyzed using ANOVA. Brackets and corresponding asterisks denote significant between-group differences: * p<0.05, ** p<0.01, *** p<0.001. Panel A: Specialists expect more than assistants, that migrant patients should adapt to institutional cultures, F(1, 49)=5.134, p=0.028. Panel B: Assistants place greater importance on knowledge of patients' backgrounds compared to specialists, F(1, 49)=12.573, p<0.001.

Our ANOVAs showed that specialists were more likely than assistants to expect that migrant patients should adapt themselves to the cultures of the institutions, F(1, 49)=5.134, p=0.028 (see Figure 2A). There were no significant differences for gender, F(2, 49)=0.650, p=0.527, urban vs. rural work settings, F(1, 49)=2.059, p=0.158, or having international professional experience, F(1, 49)=0.557, p=0.459.

Regarding the importance physicians give to the knowledge of migrant patients’ sociocultural background and histories of migration, our linear regression analysis showed that younger participants attach a higher significance to having certain knowledge of the patient's backgrounds and migration history, β=-0.411, t=-3.125, p=0.003 (see Figure 1C). This was confirmed by an ANOVA that yielded a significant difference between assistants and specialists, F(1, 49)=12.573, p<0.001, such that assistants place greater importance on knowledge of patients' socio-cultural backgrounds (see Figure 2B).

Finally, when we analyzed the associations between self-reported intercultural competence and the importance given to the knowledge of the migrant patients’ backgrounds, interestingly, we found no significant associations, β=0.007, t=0.049, p=0.961. Similarly, we found no significant associations between self-reported competence and attitudes concerning the responsibility of adaptation, β=-0.007, t=-0.046, p=0.964. As expected, we found that the importance given to knowing the migrant patients’ backgrounds was positively associated with the attitudes concerning the responsibility of adaptation, such that physicians who attached a higher level of importance to know about the migrant patients’ sociocultural backgrounds also believed more that it is the responsibility of the institutions to adapt in ways that would accommodate the cultural differences of the patients, β=0.481, t=3.804, p<0.001.

## Discussion

Our results reflected certain interesting preliminary insights on the beliefs and attitudes of Belgian emergency physicians. It was clear that most physicians find it challenging to care for migrant patients, despite expressing interest in treating this particular population. Our findings also highlight the crucial role of emergency services in providing care to migrant populations. Indeed, according to our findings, the majority of our participants report that the emergency room serves as a frontline for accessing healthcare for migrant patients and these patients frequently return to emergency departments to seek further help. Many participants also report encountering severe difficulties in the care of migrant patients, due to issues stemming from miscommunication. Nevertheless, it seems that they lack the amenities and resources necessary to facilitate smoother communication with these patients, as the majority of emergency physicians in our sample reported that they do not have access to 24/7 translation services, even on weekends. Given that previous studies are quite clear on the benefit of having translation services, especially given the cost language barriers incur, the risk of diagnostic errors, longer hospital stays, and more frequent additional tests [39-42].

The qualifications of the translators are also important, and, to our knowledge, professional translation services are not available 24/7 in Belgium, nor are they available in person. Phone translation service is available during business hours. More basic resources, such as documentation in languages other than the official language of the service (e.g., French or Dutch) or universal pictograms to facilitate basic communication, also seem to be lacking. While these measures may not eliminate all difficulties, they could enable easier and more accurate information exchange and ensure a certain level of safety in the highly specific context of acute medicine. Most participants in our sample admitted to having cared for migrant patients for reasons they did not fully understand in the first place. In light of these, ensuring a minimum level of sound communication seems to be a key and non-negotiable aspect of practicing accessible medicine for all.

Alongside the aforementioned difficulties pertaining to triage and communication, our findings highlighted other difficulties emergency physicians encounter with migrant patients, particularly patient-specific cultural barriers such as vague complaints and unrealistic expectations. The concept of vague complaints is particularly interesting given that it reflects misunderstandings in complaints expressed through a broad cultural lens, which appears to destabilize most of the physicians in our sample. The influence of culture on the way complaints are interpreted and expressed, thus, seems to have a real impact on patient care, as confirmed by Priebe and colleagues [42].

The notion of unrealistic expectations is equally noteworthy, as it suggests patients’ unfamiliarity with the Belgian healthcare system. This aspect of migrant patient care, again as highlighted previously by Priebe and colleagues [42], can pose genuine difficulties to the overall functioning of emergency services. Even when the state organizes information sessions and distributes informative documents to migrant populations, access to healthcare and ending up in the right specialization service remains a challenge for these particular groups [43].

Regarding the analyses that focused on intercultural sensitivity and competencies, our results vary from what is typically reported in the literature. We found no gender differences in the importance given to the ability to understand the psychosocial background of migrant patients, unlike Hudelson and colleagues, who report higher levels of positive attitudes in this regard among women [35], and unlike Roter and Hall, who posit that female physicians tend to practice more patient-centered communication [44]. In a similar vein, our results do not show a difference in intercultural sensitivity based on international professional experience, whereas Hudelson and colleagues find a somewhat negative effect of having worked abroad on the attitudes towards migrant patients [35], explained by the paradox that adapting to another country’s culture might lead some physicians to believe that migrants in Belgium should also adapt more easily.

Regarding the lack of a relationship between having undergone multicultural training in healthcare and a sense of intercultural competence, we remain cautious since there are no formal multicultural training programs in Belgium, and we do not know the content of the training reported by our participants.

Our analyses, however, show an increase in the sense of intercultural competence with age. It is unclear whether this results from greater experience and a better understanding of one’s own biases or whether it reflects a social desirability bias, as more senior physicians may feel more confident in their intercultural competence due to a reluctance to confront deeper cultural challenges. A systematic review of literature listed in PubMed, suggests that when intercultural competence is reported as being important, it may, in fact, reflect an overconfidence in intercultural competence and an ignorance of the true complexity of cultural issues [38]. This could suggest that those who feel most competent may lack a full understanding of the challenges posed by intercultural communication in medical contexts. Interestingly, we did not find any associations between self-reported competence and the importance given to knowledge of the migrant patients’ sociocultural backgrounds and migration histories. Nor did we find any associations between intercultural competence and the attitudes toward the responsibility of cultural adaptation in order to accommodate cultural differences. As such, self-reported measures of intercultural competence seem to warrant caution in terms of objectivity as a measure of intercultural competence.

A unique feature of our study is its evaluation of the influence of the primary practice location (i.e., rural vs. urban), which we consider particularly relevant in the Belgian context, especially given the cosmopolitan structure of its capital Brussels. Our analyses confirmed that physicians working in urban areas report higher levels of intercultural competence than those working primarily in rural areas. Seeing a larger number of migrant patients in one’s practice, however, was not associated with the levels of intercultural competence. As such, it is likely that practicing in urban settings involves other factors of influence that are yet to be researched further.

Finally, regarding physicians’ beliefs as to with whom the responsibility for adaptation lies to accommodate cultural differences, it appears that younger physicians believe more that it is the responsibility of the healthcare systems to make the necessary adjustments to ensure that migrant patients' cultural norms too are included in the institutional cultures, while older participant believes that it is up to the migrant patients to adapt to the dominant institutional culture of the healthcare system. This is consistent with the findings of Hudelson and colleagues that younger physicians and women are more likely to believe that the institutions should adapt [35]. A previous Belgian study by Dauvrin and Lorant, conducted in various medical services (but not in emergency services), demonstrated that most caregivers do not feel responsible for adapting to cultural diversity [45]. Nonetheless, the authors argue that this aspect is multidimensional and thus varies by situation. They, however, concluded that if caregivers do not feel responsible, they are less likely to have higher levels of intercultural competence [45]. Ultimately, this suggests that the self-reported levels of intercultural competence do not necessarily reflect an actual cultural sensitivity [38]. Reinforcing this trend, our results also confirm that the importance placed on patients’ psychosocial contexts and their migration histories decreases with age. Consequently, our findings lend support to the idea that self-reported intercultural competence may not be a reliable measure of actual cultural sensitivity that enables physicians to actively seek measures to improve their intercultural communication skills. It is indeed not so surprising that such complex interactions between two individuals who encounter each other for the first time in an emergency room setting, cannot be accurately reported through a subjective evaluation of confidence in one's competence.

Limitations and future directions

Despite the presence of trends both in line with and contrary to previous literature, we would like to acknowledge several limitations within our study. First and foremost, due to the specificity of our target population, our sample size of 50 complete responses remained relatively small. Additionally, since our sample only represents physicians from two Belgian regions where French is one of the official languages (i.e., Wallonia and Brussels), we did not have any responses from physicians in the Dutch-speaking region of Flanders, implying a limited generalisability of our findings to the entire Belgian context. Future studies can come up with larger samples that include physicians from multiple national and linguistic contexts (a Dutch translation of the questionnaire is available upon request from the first authors).

Secondly, discussing cultural differences can be perceived as a sensitive topic of discussion by many physicians. This social desirability bias is frequently reported in various studies, despite guarantees of anonymity and carefully formulated questionnaires. Unfortunately, such a bias tends to diminish negative attitudes toward cultural differences, which requires caution in interpreting these results, especially those relating to respondents' potential negative opinions [46]. Furthermore, due to such concerns, some potential participants may have also refused to participate in the survey despite the guaranteed anonymity and the explanations provided when distributing the questionnaire. Additionally, in the current context of economic crises or recent pandemics, the topic may be viewed as secondary or even of minor relevance by some physicians.

Another potential limitation of our study is the self-reported nature of the intercultural competence scale we incorporated within our questionnaire. Therefore, as previously mentioned, caution should be warranted when the results that involve self-evaluated intercultural competence are interpreted. Indeed, as explained earlier, self-reported intercultural competence may not reflect physicians’ true cultural sensitivity or their readiness/willingness to actively seek ways to facilitate communication with migrant patients. Our study confirms this trend and highlights the need for further qualitative studies that can provide a more nuanced evaluation of respondents' actual cultural sensitivities.

Finally, the lack of previous studies on this subject in the Belgian context and the context of emergency services in particular was a limitation while we set up our study. This led us to come up with an exploratory research design and conduct our analyses accordingly. Nevertheless, we believe that our study can serve as an initial example that can guide future studies that are geared toward these contexts specifically.

## Conclusions

All in all, the results presented in this original study conducted in the French-speaking emergency services in Belgium demonstrate the diversity of beliefs among emergency physicians in Brussels and Wallonia, concerning the care of migrant patients, while also concretizing the difficulties that are brought up by multiculturalism within the emergency services. Our work points to the growing need for structural solutions to help physicians care for migrant patients. In this regard, we believe that certain improvements aimed at facilitating communication (e.g., multilingual documentation, availability of universal pictograms, access to translation services, etc.) could enhance the work environment for emergency physicians and improve the overall quality of healthcare for migrant patients. We believe that incorporating such tools and services within the emergency care settings would benefit both patients from cultural minorities and the broader population who use emergency services. In summary, with this study, we have provided a preliminary overview of the role of cultural differences in Belgian emergency services and how the issue is interpreted and experienced by Belgian emergency physicians. As such, we hope to stimulate debate on the necessary organizational, structural, and cultural adjustments that would enable more inclusive and equitable emergency care for all.

## Appendices

Appendix A

Original French questionnaire     

**Table 2:** Original French questionnaire.

Original French questionnaire		
Questionnaire sur la prise en charge des patients migrants		
Quelques questions sur votre travail		
1) Quel pourcentage de votre temps de travail passez-vous au contact de patients (excluant administration, enseignement, recherche) ?		
Environ __________%		
2) Pouvez-vous estimer quel pourcentage de vos patients sont des migrants (patients nés et ayant grandi dans un pays autre que la Belgique) ?		
Environ __________%		
3) Quand vous voyez un patient migrant, à quelle fréquence rencontrez-vous des difficultés de communication ?		
□ Avec tous les patients migrants ou presque		
□ Avec la majorité des patients migrants (environ 3 sur 4)		
□ Avec environ la moitié des patients migrants		
□ Avec environ un patient migrant sur quatre		
□ Presque jamais		
4) Quand vous rencontrez des difficultés de communication avec des patients migrants, quelle en est l’intensité ?		
□ Difficultés très faibles		
□ Difficultés faibles		
□ Difficultés modérées		
□ Difficultés importantes		
□ Difficultés très importantes		
5) Quel niveau d’intérêt avez-vous à prendre en charge des patients migrants ?		
□ Aucun intérêt		
□ Intérêt faible		
□ Intérêt modéré		
□ Intérêt élevé		
□ Intérêt très élevé		
6) Quand les valeurs et les coutumes des migrants diffèrent de celles du pays d’accueil:		
Les institutions du pays d'accueil doivent s'adapter aux valeurs et coutumes des migrants	1 | 2 | 3 | 4 | 5 | 6 | 7	Les migrants doivent s'adapter aux institutions de leur pays d'accueil
7) Lorsque le patient ne parle pas la langue du pays (le français):		
Le médecin doit toujours mettre un interprète professionnel à disposition du patient	1 | 2 | 3 | 4 | 5 | 6 | 7	C'est la responsabilité du patient de trouver un interprète
8) Lorsque le patient manifeste une préférence à être soigné par un médecin homme ou un médecin femme :		
Les hôpitaux doivent permettre à tout patient qui en fait la demande de choisir le sexe de son médecin	1 | 2 | 3 | 4 | 5 | 6 | 7	Les patients doivent accepter d'être traités par le médecin fourni par l'hôpital, quel que soit son sexe
9) Lorsque le patient ne sait pas lire la langue du pays (le français) :		
Les hôpitaux doivent fournir des informations écrites dans la langue du patient	1 | 2 | 3 | 4 | 5 | 6 | 7	Les patients doivent s'arranger pour comprendre les informations écrites remises par l'hôpital
10) Lorsque les croyances en matière de santé du patient s’opposent aux connaissances médicales :		
Le médecin doit s'adapter aux croyances du patient concernant sa maladie et son traitement	1 | 2 | 3 | 4 | 5 | 6 | 7	Le patient doit se fier aux explications et aux recommandations du médecin
Pour bien soigner un patient migrant		
A votre avis, à quel point les éléments suivants sont-ils importants pour que le médecin puisse fournir des soins de bonne qualité à un patient migrant ?		
11) Assez de temps pour s’occuper du patient :		
1. Pas du tout d'accord		
2. Pas d'accord		
3. Ni d'accord, ni pas d'accord		
4. D'accord		
5. Tout à fait d'accord		
12) Dossier médical complet et accessible au bon moment:		
1. Pas du tout d'accord		
2. Pas d'accord		
3. Ni d'accord, ni pas d'accord		
4. D'accord		
5. Tout à fait d'accord		
13) Expérience clinique préalable avec le problème du patient:		
1. Pas du tout d'accord		
2. Pas d'accord		
3. Ni d'accord, ni pas d'accord		
4. D'accord		
5. Tout à fait d'accord		
14) Disponibilité d’un traitement efficace pour le problème du patient:		
1. Pas du tout d'accord		
2. Pas d'accord		
3. Ni d'accord, ni pas d'accord		
4. D'accord		
5. Tout à fait d'accord		
15) Connaissance des thérapies non conventionnelles (médecines alternatives ou traditionnelles):		
1. Pas du tout d'accord		
2. Pas d'accord		
3. Ni d'accord, ni pas d'accord		
4. D'accord		
5. Tout à fait d'accord		
16) Connaissance des croyances du patient concernant sa maladie:		
1. Pas du tout d'accord		
2. Pas d'accord		
3. Ni d'accord, ni pas d'accord		
4. D'accord		
5. Tout à fait d'accord		
17) Connaissance des préférences du patient concernant sa prise en charge:		
1. Pas du tout d'accord		
2. Pas d'accord		
3. Ni d'accord, ni pas d'accord		
4. D'accord		
5. Tout à fait d'accord		
18) Connaissance du contexte social et économique du patient:		
1. Pas du tout d'accord		
2. Pas d'accord		
3. Ni d'accord, ni pas d'accord		
4. D'accord		
5. Tout à fait d'accord		
19) Connaissance du contexte familial et psychologique du patient:		
1. Pas du tout d'accord		
2. Pas d'accord		
3. Ni d'accord, ni pas d'accord		
4. D'accord		
5. Tout à fait d'accord		
20) Connaissance de l’aptitude du patient à lire et à écrire:		
1. Pas du tout d'accord		
2. Pas d'accord		
3. Ni d'accord, ni pas d'accord		
4. D'accord		
5. Tout à fait d'accord		
21) Connaissance des croyances religieuses ou spirituelles du patient:		
1. Pas du tout d'accord		
2. Pas d'accord		
3. Ni d'accord, ni pas d'accord		
4. D'accord		
5. Tout à fait d'accord		
22) Connaissance générale de l’histoire et de la culture du pays d’origine du patient:		
1. Pas du tout d'accord		
2. Pas d'accord		
3. Ni d'accord, ni pas d'accord		
4. D'accord		
5. Tout à fait d'accord		
23) Connaissance du réseau des services médicaux, sociaux et associatifs destinés aux patients migrants:		
1. Pas du tout d'accord		
2. Pas d'accord		
3. Ni d'accord, ni pas d'accord		
4. D'accord		
5. Tout à fait d'accord		
24) Connaissance des conditions de départ du pays d’origine et d’arrivée dans le pays d’accueil (parcours migratoire):		
1. Pas du tout d'accord		
2. Pas d'accord		
3. Ni d'accord, ni pas d'accord		
4. D'accord		
5. Tout à fait d'accord		
25) Capacité du médecin et du patient à parler couramment la même langue:		
1. Pas du tout d'accord		
2. Pas d'accord		
3. Ni d'accord, ni pas d'accord		
4. D'accord		
5. Tout à fait d'accord		
26) Disponibilité d’interprètes:		
1. Pas du tout d'accord		
2. Pas d'accord		
3. Ni d'accord, ni pas d'accord		
4. D'accord		
5. Tout à fait d'accord		
27) Reconnaissance par le médecin de ses propres biais et préjugés à propos des patients:		
1. Pas du tout d'accord		
2. Pas d'accord		
3. Ni d'accord, ni pas d'accord		
4. D'accord		
5. Tout à fait d'accord		
Difficultés avec les patients migrants		
Parfois, les médecins rencontrent des difficultés particulières lorsqu'ils prennent en charge des patients migrants. Ces difficultés peuvent être causées par différents facteurs (voir liste ci-dessous).		
Indiquez, pour chaque facteur, s’il représente pour vous une cause rare ou fréquente de difficultés.		
28) Croyances en matière de santé du patient migrant qui s’opposent aux connaissances médicales : □		
1. Pas du tout d'accord		
2. Pas d'accord		
3. Ni d'accord, ni pas d'accord		
4. D'accord		
5. Tout à fait d'accord		
29) Attentes irréalistes du patient migrant : □		
1. Pas du tout d'accord		
2. Pas d'accord		
3. Ni d'accord, ni pas d'accord		
4. D'accord		
5. Tout à fait d'accord		
30) Connaissance insuffisante de la langue locale (français) par le patient migrant : □		
1. Pas du tout d'accord		
2. Pas d'accord		
3. Ni d'accord, ni pas d'accord		
4. D'accord		
5. Tout à fait d'accord		
31) Manque de connaissance du fonctionnement du système de santé local (Belgique) par le patient migrant : □		
1. Pas du tout d'accord		
2. Pas d'accord		
3. Ni d'accord, ni pas d'accord		
4. D'accord		
5. Tout à fait d'accord		
32) Niveau bas de scolarisation du patient migrant : □		
1. Pas du tout d'accord		
2. Pas d'accord		
3. Ni d'accord, ni pas d'accord		
4. D'accord		
5. Tout à fait d'accord		
33) Plaintes floues exprimées par le patient migrant : □		
1. Pas du tout d'accord		
2. Pas d'accord		
3. Ni d'accord, ni pas d'accord		
4. D'accord		
5. Tout à fait d'accord		
34) Manque d’expérience clinique du médecin avec certains problèmes présentés par les patients migrants : □		
1. Pas du tout d'accord		
2. Pas d'accord		
3. Ni d'accord, ni pas d'accord		
4. D'accord		
5. Tout à fait d'accord		
35) Manque de connaissances adéquates par le médecin concernant les pays et les cultures d’origine des patients migrants : □		
1. Pas du tout d'accord		
2. Pas d'accord		
3. Ni d'accord, ni pas d'accord		
4. D'accord		
5. Tout à fait d'accord		
36) Manque d’intérêt ou de motivation du médecin à prendre en charge des patients migrants : □		
1. Pas du tout d'accord		
2. Pas d'accord		
3. Ni d'accord, ni pas d'accord		
4. D'accord		
5. Tout à fait d'accord		
37) Manque de compétences par le médecin à communiquer avec des patients d’autres langues et cultures : □		
1. Pas du tout d'accord		
2. Pas d'accord		
3. Ni d'accord, ni pas d'accord		
4. D'accord		
5. Tout à fait d'accord		
38) Biais ou préjugés du médecin à l’égard des patients migrants : □		
1. Pas du tout d'accord		
2. Pas d'accord		
3. Ni d'accord, ni pas d'accord		
4. D'accord		
5. Tout à fait d'accord		
39) Utilisation des amis ou des membres de la famille comme interprètes : □		
1. Pas du tout d'accord		
2. Pas d'accord		
3. Ni d'accord, ni pas d'accord		
4. D'accord		
5. Tout à fait d'accord		
40) Mauvaise adaptation du système de santé belge aux besoins et aux attentes des patients migrants : □		
1. Pas du tout d'accord		
2. Pas d'accord		
3. Ni d'accord, ni pas d'accord		
4. D'accord		
5. Tout à fait d'accord		
41) Durée insuffisante des consultations : □		
1. Pas du tout d'accord		
2. Pas d'accord		
3. Ni d'accord, ni pas d'accord		
4. D'accord		
5. Tout à fait d'accord		
42) Manque de documents destinés aux patients traduits dans les langues des patients migrants : □		
1. Pas du tout d'accord		
2. Pas d'accord		
3. Ni d'accord, ni pas d'accord		
4. D'accord		
5. Tout à fait d'accord		
Veuillez cocher (□) les 5 causes de difficultés les plus fréquentes (« top 5 »)		
Vos compétences		
A quel point vous considérez-vous compétent(e) pour les tâches suivantes pour les patients migrants (= nés et ayant grandi dans un pays autre que la Belgique) ?		
43) Obtenir une anamnèse médicale pertinente en fonction de la plainte du patient :		
1. Pas du tout d'accord		
2. Pas d'accord		
3. Ni d'accord, ni pas d'accord		
4. D'accord		
5. Tout à fait d'accord		
44) Faire un examen clinique ciblé sur le motif de consultation du patient :		
1. Pas du tout d'accord		
2. Pas d'accord		
3. Ni d'accord, ni pas d'accord		
4. D'accord		
5. Tout à fait d'accord		
45) Obtenir une anamnèse psychosociale du patient :		
1. Pas du tout d'accord		
2. Pas d'accord		
3. Ni d'accord, ni pas d'accord		
4. D'accord		
5. Tout à fait d'accord		
46) Annoncer une mauvaise nouvelle :		
1. Pas du tout d'accord		
2. Pas d'accord		
3. Ni d'accord, ni pas d'accord		
4. D'accord		
5. Tout à fait d'accord		
47) S’assurer qu’un patient illettré comprenne le traitement de la maladie :		
1. Pas du tout d'accord		
2. Pas d'accord		
3. Ni d'accord, ni pas d'accord		
4. D'accord		
5. Tout à fait d'accord		
48) Expliquer son refus d’un examen ou d’un traitement injustifié au patient qui en fait la demande :		
1. Pas du tout d'accord		
2. Pas d'accord		
3. Ni d'accord, ni pas d'accord		
4. D'accord		
5. Tout à fait d'accord		
49) Discuter des avantages et des risques potentiels de thérapies non conventionnelles avec un patient qui les utilise :		
1. Pas du tout d'accord		
2. Pas d'accord		
3. Ni d'accord, ni pas d'accord		
4. D'accord		
5. Tout à fait d'accord		
50) Discuter des préférences et des contraintes religieuses du patient concernant son traitement :		
1. Pas du tout d'accord		
2. Pas d'accord		
3. Ni d'accord, ni pas d'accord		
4. D'accord		
5. Tout à fait d'accord		
51) Communiquer l’importance du traitement médical à un patient qui croit que sa maladie est causée par des forces surnaturelles :		
1. Pas du tout d'accord		
2. Pas d'accord		
3. Ni d'accord, ni pas d'accord		
4. D'accord		
5. Tout à fait d'accord		
52) Explorer le parcours migratoire et les expériences traumatiques éventuelles d’un requérant d’asile :		
1. Pas du tout d'accord		
2. Pas d'accord		
3. Ni d'accord, ni pas d'accord		
4. D'accord		
5. Tout à fait d'accord		
53) Travailler efficacement avec un interprète :		
1. Pas du tout d'accord		
2. Pas d'accord		
3. Ni d'accord, ni pas d'accord		
4. D'accord		
5. Tout à fait d'accord		
54) Orienter un patient migrant sans papiers vers des services médicaux et sociaux appropriés :		
1. Pas du tout d'accord		
2. Pas d'accord		
3. Ni d'accord, ni pas d'accord		
4. D'accord		
5. Tout à fait d'accord		
55) Effectuer l’examen physique d’une patiente musulmane qui porte le voile :		
1. Pas du tout d'accord		
2. Pas d'accord		
3. Ni d'accord, ni pas d'accord		
4. D'accord		
5. Tout à fait d'accord		
56) Poser les questions et donner les informations au mari de la patiente, si celle-ci le demande :		
1. Pas du tout d'accord		
2. Pas d'accord		
3. Ni d'accord, ni pas d'accord		
4. D'accord		
5. Tout à fait d'accord		
Concernant le vécu aux urgences		
Les questions suivantes portent sur ce lieu de pratique spécifique :		
57) Pensez-vous que les urgences prennent plus souvent en charge des patients migrants en comparaison aux autres spécialités médicales ?		
1. Pas du tout d'accord		
2. Pas d'accord		
3. Ni d'accord, ni pas d'accord		
4. D'accord		
5. Tout à fait d'accord		
58) Avez-vous l’impression que les patients migrants ont tendance à revenir plus facilement aux urgences par rapport aux patients nés et ayant grandi en Belgique ?		
1. Pas du tout d'accord		
2. Pas d'accord		
3. Ni d'accord, ni pas d'accord		
4. D'accord		
5. Tout à fait d'accord		
59) Pensez-vous qu’être migrant peut être à l’origine de conflits en salle d’urgence ?		
1. Pas du tout d'accord		
2. Pas d'accord		
3. Ni d'accord, ni pas d'accord		
4. D'accord		
5. Tout à fait d'accord		
60) Pensez- vous qu’il serait nécessaire d’organiser les services d’urgence afin de pouvoir mieux accueillir les patients migrants ?		
1. Pas du tout d'accord		
2. Pas d'accord		
3. Ni d'accord, ni pas d'accord		
4. D'accord		
5. Tout à fait d'accord		
61) Vous est-il déjà arrivé de ne pas comprendre le motif de consultation aux urgences d’un patient migrant et d’avoir dû poursuivre la consultation médicale en l’absence d’outil disponible pour vous aider à la compréhension ?		
1. Pas du tout d'accord		
2. Pas d'accord		
3. Ni d'accord, ni pas d'accord		
4. D'accord		
5. Tout à fait d'accord		
62) Avez-vous déjà eu l’impression qu’un patient migrant, au moment de sa sortie des urgences, ne comprenait pas vos explications concernant le diagnostic, le traitement ou le suivi proposé et ce malgré vos efforts de communication ?		
1. Pas du tout d'accord		
2. Pas d'accord		
3. Ni d'accord, ni pas d'accord		
4. D'accord		
5. Tout à fait d'accord		
63) Avez-vous l’impression de prescrire plus d’examens paracliniques aux patients migrants en comparaison aux patients nés et ayant grandi en Belgique ? (par exemple : biologies, radio, scanner etc..) :		
1. Pas du tout d'accord		
2. Pas d'accord		
3. Ni d'accord, ni pas d'accord		
4. D'accord		
5. Tout à fait d'accord		
64) Dans votre service d’urgence avez-vous accès facilement à un service de traduction efficace ?		
□ Oui	□ Non	
65) Si oui, ce service de traduction est-il disponible jour et nuit ?		
□ Oui	□ Non	
66) Dans votre service d’urgence, y-a-t-il de la documentation à destination des patients dans d’autres langues que le français ou le néerlandais ou l’allemand ?		
□ Oui	□ Non	
67) Si oui, dans quelle(s) langue(s) ?		
_____________________		
68) Avez-vous connaissance d’outils permettant une aide à la communication avec le patient migrant ? (type « Mediglotte »)		
□ Oui	□ Non	
Si oui, le(s)quel(s) ?		
_____________________		
69) Avez-vous à votre disposition dans votre service des urgences des kits de communication contenant des pictogrammes universels permettant une communication basique avec le patient migrant ?		
□ Oui	□ Non	
Quelques questions sur vous-même:		
70) Votre statut professionnel :		
□ Assistant	(année de formation : 1 - 2 - 3 - 4 - 5 - 6)	
□ Médecin		
□ Chef de service		
71) En quelle année avez-vous reçu votre diplôme de médecin urgentiste ?		
_____________________		
72) Où avez-vous obtenu votre diplôme de médecin ?		
□ Belgique		
□ A l’étranger	(pays : _____________________)	
73) Où se déroule votre activité professionnelle principale ?		
□ Zone citadine		
□ Zone rurale		
74) Vous êtes :		
□ M		
□ F		
□ X		
75) En quelle année êtes-vous né(e) ?		
___________		
76) Avez-vous la nationalité belge ?		
□ Oui	□ Non	
77) Avez-vous d’autre(s) nationalité(s) ?		
□ Oui	Spécifiez __________________________	
□ Non		
78) Dans quel pays avez-vous passé la plus grande partie de votre enfance et		
Adolescence ? (Jusqu’à l’âge de 18 ans)		
□ Belge		
□ Autre	Spécifiez __________________________	
79) Parlez-vous couramment une (ou plusieurs) langue(s) autre(s) que le français ?		
□ Oui	Spécifiez __________________________	
□ Non		
80) Parliez-vous vous le français à la maison avec vos parents quand vous étiez		
enfant ?		
□ Oui	□ Non	
81) Parliez-vous une langue autre que le français à la maison avec vos parents quand		
vous étiez enfant ?		
□ Oui	Spécifiez __________________________	
□ Non		
82) Quelle est votre religion ?		
□ Catholique		
□ Juive		
□ Musulmane		
□ Protestante		
□ Autre	Spécifiez ___________________________	
□ Aucune		
83) Avez-vous travaillé en tant que médecin ou médecin stagiaire dans un pays autre que la Belgique, pendant 6 mois ou plus ?		
□ Oui	Quel(s) pays _____________________	
□ Non		
84) Avez-vous déjà reçu une formation spécifique concernant la prise en charge de		
patients venant d’autres cultures ? (ex. colloques, séminaires, cours, stages)		
□ Oui	Spécifiez type de formation _________	
□ Non		

Appendix B

English version of the questionnaire 

**Table 3:** English version of the questionnaire.

English version of the questionnaire		
Questionnaire on the care of migrant patients		
Some questions about your work:		
1) What percentage of your working time do you spend in direct contact with patients (excluding administration, teaching, and research)?		
Approximately __________%		
2) Can you estimate what percentage of your patients are migrants (patients born and raised in a country other than Belgium)?		
Approximately __________%		
3) When you see a migrant patient, how often do you encounter communication difficulties?		
□ With almost all migrant patients		
□ With the majority of migrant patients (around 3 out of 4)		
□ With about half of migrant patients		
□ With about one in four migrant patients		
□ Almost never		
4) When you encounter communication difficulties with migrant patients, how severe are they?		
□ Very mild difficulties		
□ Mild difficulties		
□ Moderate difficulties		
□ Significant difficulties		
□ Very significant difficulties		
5) What level of interest do you have in caring for migrant patients?		
□ No interest		
□ Low interest		
□ Moderate interest		
□ High interest		
□ Very high interest		
6) When the values and customs of migrants differ from those of the host country:		
The institutions of the host country should adapt to the values and customs of migrants	1 | 2 | 3 | 4 | 5 | 6 | 7	Migrants should adapt to the institutions of their host country
7) When the patient does not speak the language of the country (French):		
The doctor should always provide a professional interpreter for the patient	1 | 2 | 3 | 4 | 5 | 6 | 7	It is the patient's responsibility to find an interpreter
8) When the patient expresses a preference to be treated by a male or female doctor:		
Hospitals should allow any patient who requests it to choose the gender of their doctor	1 | 2 | 3 | 4 | 5 | 6 | 7	Patients should accept to be treated by the doctor provided by the hospital, regardless of their gender
9) When the patient is unable to read the language of the country (French):		
Hospitals should provide written documentation in the patient's language	1 | 2 | 3 | 4 | 5 | 6 | 7	Patients should make arrangements to understand the written information provided by the hospital
10) When the patient's health beliefs conflict with medical knowledge:		
The doctor should adapt to the patient's beliefs about their illness and treatment	1 | 2 | 3 | 4 | 5 | 6 | 7	The patient should rely on the doctor's explanations and recommendations
To provide quality care to a migrant patient		
In your opinion, how important are the following elements for a doctor to provide quality care to a migrant patient?		
11) Sufficient time to take care of the patient:		
1. Strongly disagree		
2. Disagree		
3. Neither agree nor disagree		
4. Agree		
5. Strongly agree		
12) A complete medical record accessible at the right time:		
1. Strongly disagree		
2. Disagree		
3. Neither agree nor disagree		
4. Agree		
5. Strongly agree		
13) Prior clinical experience with the patient's issue:		
1. Strongly disagree		
2. Disagree		
3. Neither agree nor disagree		
4. Agree		
5. Strongly agree		
14) Availability of an effective treatment for the patient's problem:		
1. Strongly disagree		
2. Disagree		
3. Neither agree nor disagree		
4. Agree		
5. Strongly agree		
15) Knowledge of non-conventional therapies (alternative or traditional medicine):		
1. Strongly disagree		
2. Disagree		
3. Neither agree nor disagree		
4. Agree		
5. Strongly agree		
16) Knowledge of the patient's beliefs about their illness:		
1. Strongly disagree		
2. Disagree		
3. Neither agree nor disagree		
4. Agree		
5. Strongly agree		
17) Knowledge of the patient's preferences regarding their care:		
1. Strongly disagree		
2. Disagree		
3. Neither agree nor disagree		
4. Agree		
5. Strongly agree		
18) Knowledge of the patient's social and economic context:		
1. Strongly disagree		
2. Disagree		
3. Neither agree nor disagree		
4. Agree		
5. Strongly agree		
19) Knowledge of the patient's family and psychological context:		
1. Strongly disagree		
2. Disagree		
3. Neither agree nor disagree		
4. Agree		
5. Strongly agree		
20) Knowledge of the patient's ability to read and write:		
1. Strongly disagree		
2. Disagree		
3. Neither agree nor disagree		
4. Agree		
5. Strongly agree		
21) Knowledge of the patient's religious or spiritual beliefs:		
1. Strongly disagree		
2. Disagree		
3. Neither agree nor disagree		
4. Agree		
5. Strongly agree		
22) General knowledge of the history and culture of the patient’s country of origin:		
1. Strongly disagree		
2. Disagree		
3. Neither agree nor disagree		
4. Agree		
5. Strongly agree		
23) Knowledge of the network of medical, social, and community services for migrant patients:		
1. Strongly disagree		
2. Disagree		
3. Neither agree nor disagree		
4. Agree		
5. Strongly agree		
24) Knowledge of the conditions of departure from the country of origin and arrival in the host country (migration journey):		
1. Strongly disagree		
2. Disagree		
3. Neither agree nor disagree		
4. Agree		
5. Strongly agree		
25)The ability of the doctor and the patient to speak the same language fluently:		
1. Strongly disagree		
2. Disagree		
3. Neither agree nor disagree		
4. Agree		
5. Strongly agree		
26) Availability of interpreters:		
1. Strongly disagree		
2. Disagree		
3. Neither agree nor disagree		
4. Agree		
5. Strongly agree		
27) Recognition by the doctor of their own biases and prejudices about patients:		
1. Strongly disagree		
2. Disagree		
3. Neither agree nor disagree		
4. Agree		
5. Strongly agree		
Difficulties with migrant patients		
Doctors sometimes encounter specific challenges when treating migrant patients. These difficulties may be caused by various factors (see the list below).		
Please indicate, for each factor, whether it is a rare or frequent cause of difficulty for you.		
28) Migrant patient’s health beliefs conflict with medical knowledge: □		
1. Strongly disagree		
2. Disagree		
3. Neither agree nor disagree		
4. Agree		
5. Strongly agree		
29) Migrant patient’s unrealistic expectations: □		
1. Strongly disagree		
2. Disagree		
3. Neither agree nor disagree		
4. Agree		
5. Strongly agree		
30) Insufficient knowledge of the local language (French) by the migrant patient: □		
1. Strongly disagree		
2. Disagree		
3. Neither agree nor disagree		
4. Agree		
5. Strongly agree		
31) Lack of understanding of the local healthcare system (Belgium) by the migrant patient: □		
1. Strongly disagree		
2. Disagree		
3. Neither agree nor disagree		
4. Agree		
5. Strongly agree		
32) Low level of education of the migrant patient: □		
1. Strongly disagree		
2. Disagree		
3. Neither agree nor disagree		
4. Agree		
5. Strongly agree		
33) Vague complaints expressed by the migrant patient: □		
1. Strongly disagree		
2. Disagree		
3. Neither agree nor disagree		
4. Agree		
5. Strongly agree		
34) Lack of clinical experience by the doctor with certain issues presented by migrant patients: □		
1. Strongly disagree		
2. Disagree		
3. Neither agree nor disagree		
4. Agree		
5. Strongly agree		
35) Inadequate knowledge by the doctor regarding the countries and cultures of origin of migrant patients: □		
1. Strongly disagree		
2. Disagree		
3. Neither agree nor disagree		
4. Agree		
5. Strongly agree		
36) Lack of interest or motivation by the doctor to treat migrant patients: □		
1. Strongly disagree		
2. Disagree		
3. Neither agree nor disagree		
4. Agree		
5. Strongly agree		
37) Lack of skills by the doctor to communicate with patients from other languages and cultures: □		
1. Strongly disagree		
2. Disagree		
3. Neither agree nor disagree		
4. Agree		
5. Strongly agree		
38) Doctor’s biases or prejudices towards migrant patients: □		
1. Strongly disagree		
2. Disagree		
3. Neither agree nor disagree		
4. Agree		
5. Strongly agree		
39) Use of friends or family members as interpreters: □		
1. Strongly disagree		
2. Disagree		
3. Neither agree nor disagree		
4. Agree		
5. Strongly agree		
40) Poor adaptation of the Belgian healthcare system to the needs and expectations of migrant patients: □		
1. Strongly disagree		
2. Disagree		
3. Neither agree nor disagree		
4. Agree		
5. Strongly agree		
41) Insufficient duration of consultations: □		
1. Strongly disagree		
2. Disagree		
3. Neither agree nor disagree		
4. Agree		
5. Strongly agree		
42) Lack of patient documentation translated into the languages of migrant patients: □		
1. Strongly disagree		
2. Disagree		
3. Neither agree nor disagree		
4. Agree		
5. Strongly agree		
Please check (□) the 5 most frequent causes of difficulty ("Top 5").		
Your skills		
How competent do you consider yourself to be for the following tasks related to migrant patients (i.e., those born and raised in a country other than Belgium)?		
43) Obtaining relevant medical history based on the patient’s complaint:		
1. Strongly disagree		
2. Disagree		
3. Neither agree nor disagree		
4. Agree		
5. Strongly agree		
44) Conducting a targeted clinical examination based on the patient’s reason for consultation:		
1. Strongly disagree		
2. Disagree		
3. Neither agree nor disagree		
4. Agree		
5. Strongly agree		
45) Obtaining a psychosocial history of the patient:		
1. Strongly disagree		
2. Disagree		
3. Neither agree nor disagree		
4. Agree		
5. Strongly agree		
46) Delivering bad news:		
1. Strongly disagree		
2. Disagree		
3. Neither agree nor disagree		
4. Agree		
5. Strongly agree		
47) Ensuring that an illiterate patient understands the treatment for their illness:		
1. Strongly disagree		
2. Disagree		
3. Neither agree nor disagree		
4. Agree		
5. Strongly agree		
48) Explaining your refusal of an unjustified examination or treatment requested by the patient:		
1. Strongly disagree		
2. Disagree		
3. Neither agree nor disagree		
4. Agree		
5. Strongly agree		
49) Discussing the potential benefits and risks of non-conventional therapies with a patient using them:		
1. Strongly disagree		
2. Disagree		
3. Neither agree nor disagree		
4. Agree		
5. Strongly agree		
50) Discussing the patient’s religious preferences and constraints regarding their treatment:		
1. Strongly disagree		
2. Disagree		
3. Neither agree nor disagree		
4. Agree		
5. Strongly agree		
51) Communicating the importance of medical treatment to a patient who believes their illness is caused by supernatural forces:		
1. Strongly disagree		
2. Disagree		
3. Neither agree nor disagree		
4. Agree		
5. Strongly agree		
52) Exploring the migration journey and any traumatic experiences of an asylum seeker:		
1. Strongly disagree		
2. Disagree		
3. Neither agree nor disagree		
4. Agree		
5. Strongly agree		
53) Working effectively with an interpreter:		
1. Strongly disagree		
2. Disagree		
3. Neither agree nor disagree		
4. Agree		
5. Strongly agree		
54) Referring an undocumented migrant patient to appropriate medical and social services:		
1. Strongly disagree		
2. Disagree		
3. Neither agree nor disagree		
4. Agree		
5. Strongly agree		
55) Conducting a physical examination of a Muslim woman wearing a veil:		
1. Strongly disagree		
2. Disagree		
3. Neither agree nor disagree		
4. Agree		
5. Strongly agree		
56) Asking questions and providing information to the patient’s husband, if she requests it:		
1. Strongly disagree		
2. Disagree		
3. Neither agree nor disagree		
4. Agree		
5. Strongly agree		
Regarding experiences in emergency departments		
The following questions concern this specific area of practice:		
57) Do you think emergency departments treat migrant patients more frequently compared to other medical specialties?		
1. Strongly disagree		
2. Disagree		
3. Neither agree nor disagree		
4. Agree		
5. Strongly agree		
58) Do you feel that migrant patients tend to return to the emergency department more often than patients born and raised in Belgium?		
1. Strongly disagree		
2. Disagree		
3. Neither agree nor disagree		
4. Agree		
5. Strongly agree		
59) Do you think being a migrant can cause conflicts in the emergency department?		
1. Strongly disagree		
2. Disagree		
3. Neither agree nor disagree		
4. Agree		
5. Strongly agree		
60) Do you think it would be necessary to organize emergency services to better accommodate migrant patients?		
1. Strongly disagree		
2. Disagree		
3. Neither agree nor disagree		
4. Agree		
5. Strongly agree		
61) Have you ever found yourself unable to understand the reason for consultation of a migrant patient in the emergency department and had to proceed without any tools available to aid comprehension?		
1. Strongly disagree		
2. Disagree		
3. Neither agree nor disagree		
4. Agree		
5. Strongly agree		
62) Have you ever felt that a migrant patient, upon discharge from the emergency department, did not understand your explanations regarding the diagnosis, treatment, or follow-up despite your communication efforts?		
1. Strongly disagree		
2. Disagree		
3. Neither agree nor disagree		
4. Agree		
5. Strongly agree		
63) Do you feel that you prescribe more diagnostic tests (e.g., bloodwork, X-rays, CT scans, etc.) for migrant patients compared to patients born and raised in Belgium?		
1. Strongly disagree		
2. Disagree		
3. Neither agree nor disagree		
4. Agree		
5. Strongly agree		
64) In your emergency department, do you have easy access to an effective translation service?		
□ Yes	□ No	
65) If yes, is this translation service available 24/7?		
□ Yes	□ No	
66) In your emergency department, is there documentation available for patients in languages other than French, Dutch, or German?		
□ Yes	□ No	
67) If yes, in which language(s)?		
___________________________________		
68) Are you aware of tools that assist communication with migrant patients (e.g., "Mediglotte")?		
□ Yes	□ No	
If yes, which one(s)?		
______________________________________		
69) Does your emergency department have communication kits with universal pictograms for basic communication with migrant patients?		
□ Yes	□ No	
A few questions about yourself		
70) Your professional status:		
□ Assistant	(year of training: 1 - 2 - 3 - 4 - 5 - 6)	
□ Doctor		
□ Head of Department		
71) In which year did you receive your emergency medicine degree?		
_____________		
72) Where did you obtain your medical degree?		
□ Belgium		
□ Abroad	(country: _____________________)	
73) Where is your primary professional activity located?		
□ Urban area		
□ Rural area		
74) You are:		
□ M		
□ F		
□ X		
75) What year were you born?		
___________		
76) Do you have Belgian nationality?		
□ Yes	□ No	
77) Do you have another nationality or other nationalities?		
□ Yes	Specify __________________________	
□ No		
78) In which country did you spend most of your childhood and adolescence (up to age 18)?		
□ Belgium		
□ Other	Specify __________________________	
79) Do you speak fluently a language other than French?		
□ Yes	Specify __________________________	
□ No		
80) Did you speak French at home with your parents when you were a child?		
□ Yes	□ No	
81) Did you speak a language other than French at home with your parents when you were a child?		
□ Yes	Specify __________________________	
□ No		
82) What is your religion?		
□ Catholic		
□ Jewish		
□ Muslim		
□ Protestant		
□ Other	Specify __________________________	
□ None		
83) Have you worked as a doctor or medical trainee in a country other than Belgium for 6 months or more?		
□ Yes	Which country/countries? __________	
□ No		
84) Have you ever received specific training related to the care of patients from other cultures (e.g., conferences, seminars, courses, internships)?		
□ Yes	Specify the type of training _________	
□ No		
